# Physiotherapeutic Reduction of Orofacial Pain Using Extremely Low-Frequency Electromagnetic Field and Light-Emitting Diode Therapy—A Pilot Study

**DOI:** 10.1155/2022/3115154

**Published:** 2022-02-08

**Authors:** Danuta Lietz-Kijak, Roman Ardan

**Affiliations:** ^1^Department of Propaedeutic Physical Diagnostics and Dental Physiotherapy, Faculty of Medicine and Dentistry, Pomeranian Medical University, Szczecin, Poland; ^2^Department of Econometrics, Faculty of Economic Sciences, Koszalin University of Technology, Koszalin, Poland

## Abstract

**Introduction:**

Pain is a natural response of the body to injury and one of the symptoms defining an inflammatory reaction. It is almost always present after orthognathic surgeries (OGS), but its severity is subjective in each patient. Postoperative care of the patient is aimed at minimizing of postoperative pain relief orofacial region. Options of physiotherapy include extremely low-frequency electromagnetic field (ELF EMF) and high-energy light-emitting diode (LED). *Aim of the Study*. The aim of this study was to evaluate the effects of physiotherapy combining ELF EMF and LED to reduce pain of the orofacial region in patients after OGS. *Material and Methods*. The study was conducted in thirty-two patients who underwent OGS to treat morphological defects. The participants were randomly divided into two groups: Physiotherapy group (PT) and Control group (CG). In both groups, patients were prescribed Paracetamol and nonsteroidal analgesics (NSAID—ibuprofen). Patients from the PT group additionally received postoperative physiotherapy immediately after leaving the surgical clinic in the form of ELF EMF and LED therapy. Physiotherapeutic treatments were performed for 10 days, three applications a day, at no cost to the patient. Pain intensity was assessed using the visual analogue scale (VAS), which is a reliable instrument for the measurement of pain intensity self-reported by the patient.

**Results:**

Faster reduction of pain was the major observation made in patients who received physiotherapy treatments. In all subjects, after 5 days of therapy, the pain intensity was reduced by about 50% or resolved completely. Effects of therapy were measured with the relative changes in the pain intensity score, showing what fraction of the initial pain was eliminated at the first stage and throughout the whole therapy. The analysis of relative changes instead of absolute changes allowed us, among other things, to eliminate the bias of the higher initial pain intensity in the CG group compared to the PT group.

**Conclusions:**

The conducted research revealed that the combined use of ELF EMF and LED is beneficial in the reduction of pain of patients after OGS. The analgesic effects of physiotherapy in the treatment after OGS are necessary to continue research in this area and analyze the possibility of extending the indications for its use in other surgically treated maxillofacial diseases.

## 1. Introduction

The correction of orthognathic deformities requires interdisciplinary orthodontic, surgical, and rehabilitation treatment. The preoperative orthodontic preparatory procedure is aimed at aligning the dental arches and lasts for 6 to 24 months, depending on the type and degree of the deformity. It involves the repositioning of teeth, regardless of their occlusal relations to the opposing arch, which leads to the decompensation and aggravation of the existing defect. This procedure enables the proper fusion of osteotomized fragments and correction of malocclusion during surgery. The surgical techniques that have evolved over recent decades now allow for almost any type of repositioning within the facial bone structure. Orthognathic surgeries (OGS) may concern a single jaw, either maxilla (Le Fort I type osteotomy) or mandible (bilateral sagittal split osteotomy—BSSO), or both jaws, when both techniques are combined during one procedure [1, 2]. OGS is frequently used to correct skeletal classes II and III deformities, dentofacial maxillary deformities, mandibular laterognathism, and maxillofacial asymmetry [3, 4]. Each year 234.2 million major surgeries are carried out worldwide [5]. As with any other surgical procedure, various intraoperative and postoperative complications may occur in patients undergoing OGS. Most patients experience mild to severe pain. Postoperative pain management is very important to reduce the stress caused by the onset of pain, to prevent instability of the circulatory system [6], to restore normal respiratory function, and to ensure fast recovery [7]. Postoperative pain is usually controlled with opioids, which are popular in the United States [8]. Major surgeries are associated with the risk of many early and late complications. Early complications, developing within the first 24 hours postoperatively, include orofacial pain, bleeding, ventilation disorders, soft tissue oedema, inflammatory reactions, infections, nausea, and vomiting. Late complications may occur at different times after surgery and include the recurrence of the defect, unfavourable nasolabial aesthetics, nasal septum deformity, temporomandibular joint dysfunction, idiopathic condylar atrophy, oral or vestibulo-nasal fistulas, osteonecrosis, and neurological disorders [9].

Postoperative care of the patient is aimed at minimizing the risk of complications and treatment of existing ones. Directly after the surgery it includes the monitoring of basic vital parameters, preventing the development of infection, reducing pain and oedema, maintaining the proper nutritional status and hydration of the patient, and psychological support. Pharmacotherapy relies on antibiotics and analgesics, usually nonsteroidal and steroidal anti-inflammatory drugs [10–12]. Stimulating treatment includes cold compresses to reduce pain and oedema. Pain is almost always present after OGS, but its severity is subjective in each patient. Incidentally, the lack of, or only minor, pain within the lower facial region may indicate functional disorders of the inferior alveolar nerve [13, 14].

In all diseases and complications of the stomatognathic system, regardless of the severity of pain, its control is among the major tasks in care of the patient. The physical and mental comfort of patients can be restored with physiotherapy and devices with extremely low-frequency electromagnetic field (ELF EMF) and light-emitting diode (LED) or low level laser therapy (LLLT) [15]. This fact inspired the authors to undertake research on the use of physiotherapy in patients after OGS in order to reduce pain of the orofacial region [16, 17].

## 2. Objective

The aim of this study was to evaluate the effects of physiotherapy combining ELF EMF and LED to reduce pain of the orofacial region in patients after OGS.

## 3. Materials and Methods

### 3.1. Patients

The study was conducted in thirty-two patients of both genders (26 female and 6 male), aged 19–24 years (mean 21.2, SD 1.44), who underwent OGS on between October 2015 and June 2017. All subjects qualified for the study underwent the same surgery—BSSO. The participants were randomly divided into two groups. In the PT group (16 patients–14 female and 2 male), mean age was 20.9, SD 1.26 years. In the control group CG (16 patients – 12 females and 4 males), mean age was 21.4, SD 1.59 years. In both groups, patients were prescribed Paracetamol and nonsteroidal analgesics (NSAID—ibuprofen). Paracetamol was used 1000 mg daily in doses four times a day, while Ibuprofen 400 mg three times a day. Patients from the PT group received postoperative physiotherapy immediately after their discharge from the surgical clinic (day 2 after surgery). Patients from the control group CG did not receive physiotherapy but only did receive analgesics. Pain severity was assessed using the visual analogue scale (VAS), which is a reliable instrument for the measurement of pain intensity self-reported by the patient. Pain intensity was measured on days 1, 5, and 10 after the beginning of therapy.

Study participants met the following inclusion criteria: general good health, lack of pre-existing medical conditions, and no medication that would affect their eligibility for surgery or compromise wound healing after surgery. Each patient was operated on by the same maxillofacial surgeon, who used the same surgical technique on both sides of the mandible to minimize differences in the functional performance of the oral tissues. We excluded patients on regular drug therapy, and those with mental illness, coagulopathy, diabetes, and chronic infections. None of the subjects was addicted to nicotine, alcohol, or illegal drugs.

The study registered on clinicaltrials.gov carries the number KB-0012/149/15. The study complied with ethical standards, and all participants signed a written informed consent form and were informed about the technique and course of the research. Participants in the study did not receive any financial incentive and could withdraw from the study at any time.

### 3.2. Devices and Parameters of Therapy

**Figure 1:**
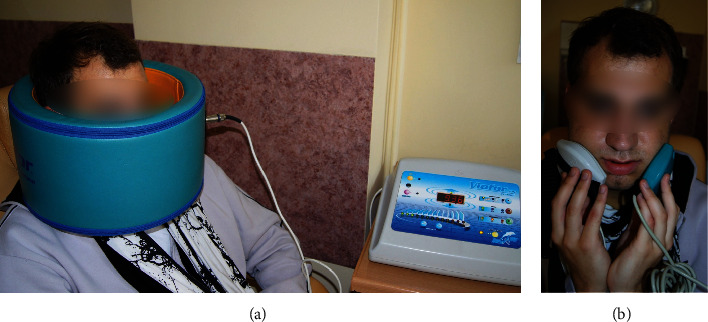
(a) and (b). Treatments with an ELF EMF, ring applicator, and elliptic applicators.

Physiotherapy was carried out using a VIOFOR JPS device (Med & Life- Poland) that produced the ELF EMF by the method М2, programme P3, and intensity 6. This means that the frequency was within the range of 180–195 Hz for the basic impulse, 12.5 Hz-29 Hz for impulse packets, 2.8–7.6 Hz for groups of packets, and 0.08–0.3 Hz for the series. The intensity of ELF EMF was set at 6, meaning the successful induction of an electromagnetic field equal to 15 *μ*T. Treatments with ELF EMF were each time provided with a ring applicator fitted onto the patient's head and generating a uniform field, and elliptic applicators used topically (Figures 1(a) and 1(b)).

**Figure 2:**
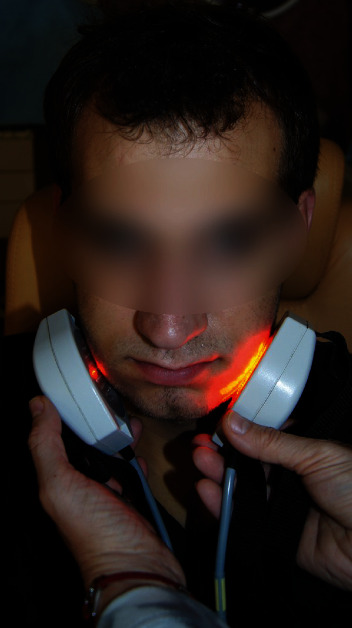
Applicators generating an extremely ELF EMF and light energy (830 nm and 640 nm) emitted from the LEDs during the physiotherapy treatment.

Patients after OGS, in the PT group, were treated with physiotherapy combining ELF EMF and LED therapy. Treatments with LED (Figure 2) were done with a device set at the M1P3 programme, which ensures constant application of the selected light intensity, and uses the highest values of ion cyclotron resonance, stimulated inside the cells. Patients received physiotherapy once a day, and this consisted of three above-described treatments for a period of 10 days.

## 4. Results

**Figure 3:**
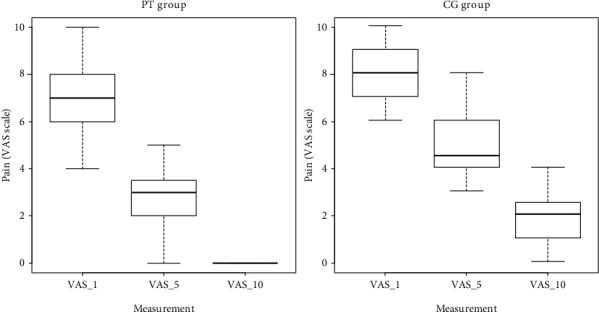
Reduction in pain intensity after physiotherapy treatments with ELF EMF and LED (PT group vs CG group).

Faster reduction of pain was the major observation made in patients who received physiotherapy treatments. In most cases, after the first day of physiotherapy, patients discontinued medication with analgesics. It should be noted that in the group receiving physiotherapy only, one patient reported pain score 10 on the VAS scale immediately after OGS. This patient also discontinued pain-controlling medication after the first day of physiotherapy. Measured intensities of pain on days 1, 5, and 10 are marked VAS_1_, VAS_5_, and VAS_10_, respectively. Descriptive statistics for the group receiving physiotherapy (PT) and the control group (CG) are presented in Table 1. Distribution of variables is presented in Figure 3.

### 4.1. Analysis of Effects of Physiotherapy (PT) and Pharmacotherapy (CG)

Because of the zero value and lack of variability in the measurement of VAS_10_ in the PT group, the reduction of pain intensity after physiotherapy treatments was analyzed by testing the statistical significance of mean variables VAS_1_ and VAS_5_ and the statistical significance of differences between these means. Results from statistical tests are presented in Table 2.

The study demonstrated reduction in pain intensity after physiotherapy at both stages of therapy (days 1–5, 5–10) and for the whole therapy (days 1–10).

In the CG group (only pharmacotherapy), the reduction of pain intensity was analyzed with repeated measures ANOVA. Measurements did not differ significantly from normal distribution (Shapiro–Wilk test, *p* > 0.05 for all three VAS measurements), and there were no significant differences in the variance of measurements (Levene test, *p* > 0.05).

The study demonstrated reduced pain intensity in the CG. The post hoc analysis of variance (Tukey HSD test) revealed significant differences between all measurements (Table 3).

### 4.2. Comparison of Effects of Physiotherapy (PT) and Pharmacotherapy (CG)

Effects of therapy were measured with the relative changes in the pain intensity score, showing what fraction of the initial pain was eliminated at the first stage and throughout the whole therapy. The analysis of relative changes instead of absolute changes allowed us, among other things, to eliminate the bias of the higher initial pain intensity in the CG group compared to the PT group.

(1) $$\begin{array}{c} {\text{Change}}_{1}=\frac{{\text{VAS}}_{5}-{\text{VAS}}_{1}}{{\text{VAS}}_{1}},\\ {\text{Change}}_{1,2}=\frac{{\text{VAS}}_{10}-{\text{VAS}}_{1}}{{\text{VAS}}_{1}}. \end{array}$$

The adopted variables showed significantly different variances in both groups, so the mean values of variables were compared with the Welch *t*-test. Results are presented in Table 4.

**Figure 4:**
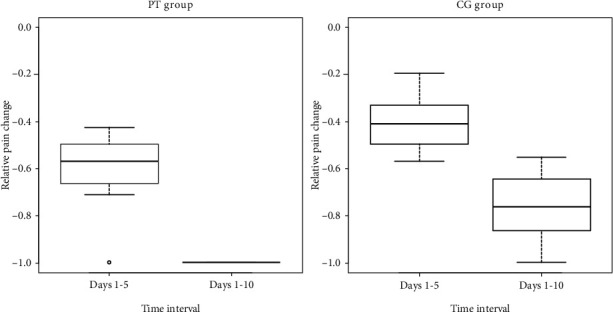
Distribution of relative changes in pain intensity in the PT and CG (lower = greater pain relief).

The study demonstrated that physiotherapy (PT) provided significantly better outcomes compared to pharmacotherapy in the (CG). This advantage was achieved as early as at the first stage, so physiotherapy reduced pain-related complaints. The distribution of relative changes in pain intensity is presented in Figure 4.

## 5. Discussion

Pain is one of the symptoms defining inflammatory reaction and also the major symptom for which most patients seek medical advice [18]. Pain is almost always present after OGS, but its severity is subjective in each patient. Regardless of the severity of pain, its control is among the major tasks in the postoperative care of the patient.

In own research it was proved that reduction of pain was the major observation made in patients who received physiotherapy treatments. The study demonstrated that physiotherapy-PT group provided significantly better outcomes compared to pharmacotherapy in the CG group. This advantage was achieved as early as at the first stage, so physiotherapy reduced pain-related complaints. Effects of therapy were measured with the relative changes in the pain intensity score, showing what fraction of the initial pain was eliminated at the first stage and throughout the whole therapy. The analysis of relative changes instead of absolute changes allowed us, among other things, to eliminate the bias of the higher initial pain intensity in the CG group compared to the PT group.

The limitations of this study concerned a small research group and the inability to divide into male and female subgroups, which would undoubtedly extend the statistical analysis and increase the value of the research. It should be noted that the manuscript presents the research group described as a preliminary report, and the research is continuing.

The analgesic and anti-inflammatory effects of ELF EMF have been well documented. Thomas applied that ELF EMF in the treatment of chronic musculoskeletal pain and proved its analgesic effect [19]. Arneja et al. used ELF EMF in the treatment of chronic lower back pain in patients with degenerative disc disease. His pilot study suggests that the ELF EMF treatment protocol is clinically relevant and can be used as a safe and effective method [20]. Pall concluded that the direct effect of ELF EMF is achieved via the activation of calcium channels, transmembrane transport of ions, and increased the activity of enzymes [21]. The analgesic effect of ELF EMF is mainly due to the increased secretion of endogenous opioid neuropeptides from the class of *ß*-endorphins, responsible for elevated pain threshold. Regenerative effects primarily come from the intensified use of oxygen and tissue respiration associated with increased diffusion and oxygen uptake by haemoglobin and cytochromes. Increased oxygen uptake stimulates tissue respiration and DNA synthesis and accelerates the mitotic cycle. The anti-inflammatory effect is associated with the stimulation of c-AMP and prostaglandin E synthesis, which affects the accumulation of c-AMP, and reduces the secretion of inflammatory mediators from neutrophils, basophils, mast cells, and lymphocytes. Iannitti conducted a clinical study in elderly people using ELF EMF and reported significant benefit in terms of reduced pain and stiffness of joints and improved physical function [22]. Nelson concluded that noninvasive ELF EMF therapy causes rapid and significant pain reduction in the early stage of knee osteoarthritis [23].

Physiotherapy with ELF EMF and LED used in own research is an innovative method that relies on the combined use of light energy emitted from high-energy diodes and an extremely low-frequency electromagnetic field. This light is monochromatic, i.e., all photons have the same wavelength, and collimated, which means it has parallel rays without divergence. However, this light is not coherent (ordered), i.e., not all the photons have a constant phase, which makes it different from laser light. Calderhead confirmed that the energy of the light generated by LEDs, used predominantly in aesthetic medicine, has a mainly local effect on tissues and can penetrate into their deeper layers, depending on the length of the generated waves and the angle of incidence [24]. Barolet employed LEDs in dermatology and used the reaction of a tissue dependent on energy absorption in specific skin layers. The effects of 830 nm infrared light (IR) are initiated at the level of the cell membrane and the effects of 640 nm red light (R) in the mitochondria. Studies on the mechanisms of action of LEDs indicated many aspects that can be considered to achieve clinical benefits. For example, LEDs influence cell metabolism by stimulating intracellular photobiochemical reactions. Different effects are observed, including increased synthesis of ATP, modification of reactive oxygen species, induction of transcription factors, changes in collagen synthesis, stimulation of angiogenesis, and enhanced blood flow [25]. Other studies demonstrated that red LEDs activate fibroblast growth factor, increase the level of type 1 procollagen, increase the level of matrix-metallo-proteinase-9 (MMP-9), and reduce the level of MMP-1, as confirmed in vitro by Barolet et al. [26]. Other researchers believe that monochromatic near-infrared light stimulates blood circulation by inducing the release of guanylate cyclase and nitrous oxide, which in turn promotes vasodilation and growth, as well as angiogenesis, leading to wound healing [27]. Simpson et al. reported that near-infrared LEDs offer the deepest penetration of the tissues with visible wavelengths and are therefore used for therapies targeted at subcutaneous structures and fibroblasts [28]. Red LEDs have been investigated in a wide range of applications, including wound healing, treatment of precancerous lesions, warts, and the prevention of inflammations of the oral mucosa. IR light emitted by LEDs can penetrate 5 to 10 mm deep into the skin and has been used for the treatment of wounds, ulcerations, and cutaneous scleroderma and was also effective in the treatment of cellulite [29–31]. Studies by Russell et al. demonstrated that the exposure of patients to a combination of different wavelengths emitted by LEDs was more effective compared to monotherapy [32]. A prospective, placebo-controlled, double-blind clinical study on the use of LEDs emitting red (640 nm) and IR light (830 nm) was performed by Lee et al. [33]. A significant smoothing of wrinkles with improved skin elasticity was observed in patients from all study groups. Tissue tests revealed a significant increase in the content of collagen and elastic fibres near highly active fibroblasts. The levels of proinflammatory cytokine, interleukin 1*β* (IL-1*β*), and tumour necrosis factor *α* (TNF-*α*) were increased, while the level of interleukin 6 (IL-6) was decreased. In another study, Goldberg et al. investigated the effects of combined red LED (633 nm) and IR (830 nm) on the skin and reported smoothing of periorbital wrinkles in 80% of patients. Histological examination showed an increased number and thickness of collagen fibres [34]. Similar studies carried out by Tian et al. in 2012 demonstrated an increased expression of type 1 collagen and the number of viable fibroblasts after treatment with different combinations of 630 nm, 830 nm, and various wavelengths of red and IR light [35].

The effect of ELF EMF in the management of postoperative pain, used in the above studies, can be explained by the cyclotron resonance known from magnetobiology and the influence of electromagnetic fields on the concentration of calcium ions and activation of nitric oxide synthetase. Animal studies have demonstrated that damage to the peripheral nerves and consequent activation of induced nitric oxide synthetase are important in the pathogenesis of neuropathic pain and the development of hyperalgesia. The analgesic effect observed in own research, induced by ELF EMF in cells after the use of the P3 programme, can be explained based on the model of ion cyclotron resonance, described by Brustkern, Graham and Leach et al. [36–38]. According to the theory, using this model in magnetobiology, calcium ions are the main target of the electromagnetic field within cell membranes and cytosol. Studies on the activity of membrane enzymes and transport of calcium ions induced by ELF EMF have indicated changes in intracellular calcium concentrations. Calcium concentration influences changes in the polarization of the cell membrane towards long-term synaptic enhancement or weakening, and thus determines the increase or decrease of cell reactivity. Change in the perception of pain following the treatment with ELF EMF and LED in patients after OGS may be related to this process, but further research is necessary to explain these mechanisms [39].

## 6. Conclusion

The results of own study on the effects of combined treatment with ELF EMF and LED demonstrated that this method provides benefits in the reduction of pain in orofacial region of patients after OGS. Combined physiotherapy treatments allowed for significantly greater pain relief compared to pharmacotherapy. Patients also resumed their social and professional activity in a shorter time after surgeries. Research in this area should be continued to analyze the possibility of extending the use of this therapeutic modality in other diseases treated by maxillofacial surgery.

## Figures and Tables

**Table 1:** Descriptive statistics for pain intensity.

Variable	Min	Max	Mean ± SD
*PT group*			
VAS_1_	4	10	7.125 ± 1.500
VAS_5_	0	5	2.875 ± 1.147
VAS_10_	0	0	0 ± 0.000

*CG group*			
VAS_1_	6	10	8.062 ± 1.237
VAS_5_	4	8	4.812 ± 1.424
VAS_10_	0	4	1.938 ± 1.289

**Table 2:** Significance of mean differences in pain intensity for the PT group.

Variable	Mean	*t*	95% CI	*p*
*PT group*				
VAS_1_	7.125	19.0	(6.326, 7.924)	≤0.001
VAS_5_	2.875	10.0	(2.264, 3.486)	≤0.001
VAS_1_–VAS_5_	4.25	13.2	(3.562, 4.938)	≤0.001

**Table 3:** Results of Tukey HSD test for the CG group.

Difference	Mean	95% CI	*p*
*CG group*			
VAS_1_–VAS_10_	6.125	(4.995, 7.255)	≤0.001
VAS_5_–VAS_10_	2.875	(1.745, 4.005)	≤0.001
VAS_1_ VAS_5_	3.250	(2.120, 4.380)	≤0.001

**Table 4:** Significance of differences in mean changes in pain intensity between groups.

Variable	Mean PT	Mean CG	*t*	95% CI for difference	*p*
Change_1_	–0.597	–0.411	–4.2	(–0.278, –0.094)	≤0.001
Change_1,2_	–1.000	–0.769	–6.8	(–0.304, –0.158)	≤0.001

## Data Availability

The data sets used to support the findings of this study are available from the corresponding author upon request.

## Abbreviations

OGS: Orthognathic surgery
BSSO: Bilateral sagittal split osteotomy
ELF EMF: Extremely low-frequency electromagnetic field
LED: Light-emitting diode
IR: Infrared light
R: Red light.
